# Synthesis of three-dimensional calcium carbonate nanofibrous structure from eggshell using femtosecond laser ablation

**DOI:** 10.1186/1477-3155-9-1

**Published:** 2011-01-20

**Authors:** Amirhossein Tavangar, Bo Tan, Krishnan Venkatakrishnan

**Affiliations:** 1 Department of Mechanical and Industrial Engineering, Ryerson University, 350 Victoria Street, Toronto, ON M5B 2K3, Canada; 2 Department of Aerospace Engineering, Ryerson University, 350 Victoria Street, Toronto, ON, M5B 2K3, Canada

## Abstract

**Background:**

Natural biomaterials from bone-like minerals derived from avian eggshells have been considered as promising bone substitutes owing to their biodegradability, abundance, and lower price in comparison with synthetic biomaterials. However, cell adhesion to bulk biomaterials is poor and surface modifications are required to improve biomaterial-cell interaction. Three-dimensional (3D) nanostructures are preferred to act as growth support platforms for bone and stem cells. Although there have been several studies on generating nanoparticles from eggshells, no research has been reported on synthesizing 3D nanofibrous structures.

**Results:**

In this study, we propose a novel technique to synthesize 3D calcium carbonate interwoven nanofibrous platforms from eggshells using high repetition femtosecond laser irradiation. The eggshell waste is value engineered to calcium carbonate nanofibrous layer in a single step under ambient conditions. Our striking results demonstrate that by controlling the laser pulse repetition, nanostructures with different nanofiber density can be achieved. This approach presents an important step towards synthesizing 3D interwoven nanofibrous platforms from natural biomaterials.

**Conclusion:**

The synthesized 3D nanofibrous structures can promote biomaterial interfacial properties to improve cell-platform surface interaction and develop new functional biomaterials for a variety of biomedical applications.

## Background

Autogenous bone has long been considered the ideal grafting material in bone reconstructive surgery owing to its osteogenic, osteoinductive and osteoconductive properties [1,2]. However, harvesting the autogenous bone requires an additional surgery which increases morbidity at the donor site and extends the operation period [3,4]. Therefore, a variety of new bone grafting materials has substituted for autogenous grafts thanks to recent advances in biotechnology. Among them, natural bone substitute biomaterials from bovine sources and bone-like minerals (calcium carbonate) derived from corals or avian eggshells, have been preferred due to their biodegradability, abundance and lower price in comparison with synthetic biomaterials [5-9]. The coralline calcium carbonate (calcite), which is totally resorbable and biocompatible and shows good osteoconductivity, has been used as an effective bone substitute in the natural form or converted to hydroxyapatite (HA) in bone healing in dentistry and orthopedic [4,10-14].

Avian eggshell, with a mineral composition similar to corals, has been introduced as a potential bone substitute in maxillodacial and craniofacial surgery as they could easily be acquired and contain ions of Sr and F [4,15] and [16]. One of the crucial characteristics to be considered when using a bone substitute graft is its degradation rate due to the fact that it may have effects on the long-term results. The graft should undergo only minimal resorption if it is used as an onlay graft whereas a resorbable one is desirable when a bone substitute is used as interpositional graft or in a peri-implant defect [15]. Eggshell, which can be manufactured under powdered or block form, can be used for both indications.

Many *in vitro *and *in vivo *studies have shown that the microporous surface structure and biodegradability of bone substitutes play critical roles in bone healing. It is indicated that cell attachment and proliferation are improved on nanostructure surface than microstructure one [17].

Among the nanoscale structures, randomly interwoven nanofibrous structures are particularly preferred for scaffolding systems in comparison with nanoparticles due to their continuous structure. The vantage of a surface comprised of ultra-fine, continuous nanofibers would be high porosity, high surface volume ratio, variable pore-size distribution, and first and foremost, morphological similarity to natural Extra Cellular Matrix (ECM) [18]. There are reported studies where eggshell has been used to compose different Ca-precursor nanoparticles or HA nano-powder that requires the additional step such as sintering to synthesize porous surfaces [16,19]. Whereas, no studies on synthesizing 3D nanofibrous structure on natural biomaterials have been accounted so far. Therefore, a simple method to generate 3D nanofibrous structure in a single-step would be in a great interest.

In the presented work, we have proposed a novel technique to synthesize calcium carbonate 3D nanofibrous structures from eggshells using femtosecond laser processing. To the best of authors' knowledge, this is the first work on synthesizing 3D calcium carbonate nanofibrous structures using femtosecond laser. We also have investigated the effects of laser pulse repetition on the density of nanofibers and the structure pore size.

## Results and discussion

**Figure 1:**
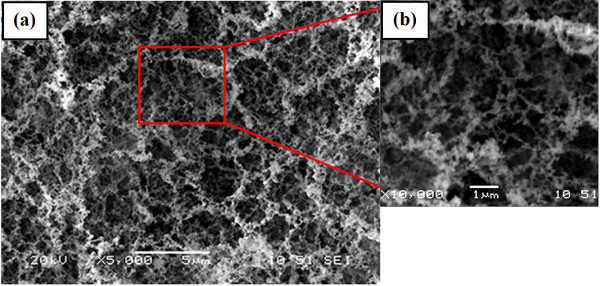
**SEM images of calcium carbonate nanofibrous structure synthesized on an eggshell at laser repetition rate of 4 MHz at magnification of a) X5000 and b) X10000**.

**Figure 2:**
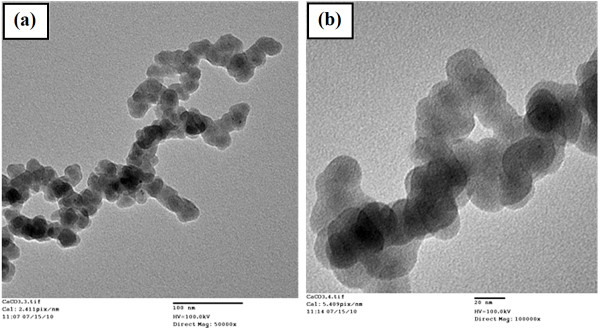
**TEM images of calcium carbonate nanofibers synthesized at repetition rate of 4 MHz at magnification of a) X50000 and b) X100000**.

The morphology of the nanofibrous structures is influenced by various laser parameters such as, laser fluence, laser repetition, and laser dwell time. In this study, we investigate the effect of laser repetition on porosity of the structures. Figure. 1 shows the nanofibrous structure generated at repetition of 4 MHz. A close-up view of the structures shows that they consist of self-assembled closed-rings and bridges in which particles are fused together, as shown in Figure. 1. TEM image of a single nanofiber demonstrates a high degree of nanoparticle aggregation with average size of 50 ± 20 nm (Figure. 2). Therefore, the bond between the particles themselves and with the eggshell substrate is assumed to be strong.

**Figure 3:**
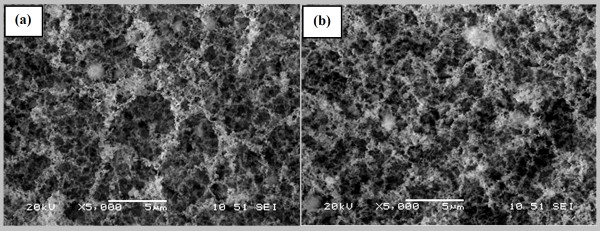
**SEM images of calcium carbonate nanofibrous structure synthesized on an eggshell at laser repetition rates of a) 8 MHz, and b) 13 MHz**.

Further experiments have been performed with different laser repetition rates at 8 and 13 MHz (see Figure. 3). Comparing Figures 3a and 3b, it can be observed that the structure pore size has been decreased by increasing the repetition rate. This is due to the increase in density of synthesized nanofibers.

**Figure 4:**
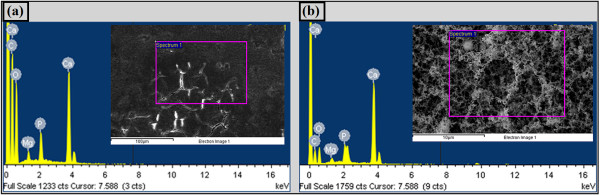
**Energy-dispersive X-ray spectroscopy (EDS) analyses of a) unprocessed eggshell, and b) synthesized calcium carbonate nanofibrous structure**.

The Energy-dispersive X-ray spectroscopy (EDS) analysis, an integrated feature of a SEM, has been conducted in order to evaluate the composition of nanofiberous structure. Figure. 4 depicts the EDS analyses which compare the elemental composition of nanofibrous structures with an unprocessed eggshell. Although all the elements presented on the unprocessed eggshell, i.e., Ca, P, Mg, C, and O, can be recognized on the synthesized nanofibrous structure, the percentage of oxygen and carbon has been decreased significantly in the nanofibers which implies the decomposition of the organic part due to laser irradiation.

**Figure 5:**
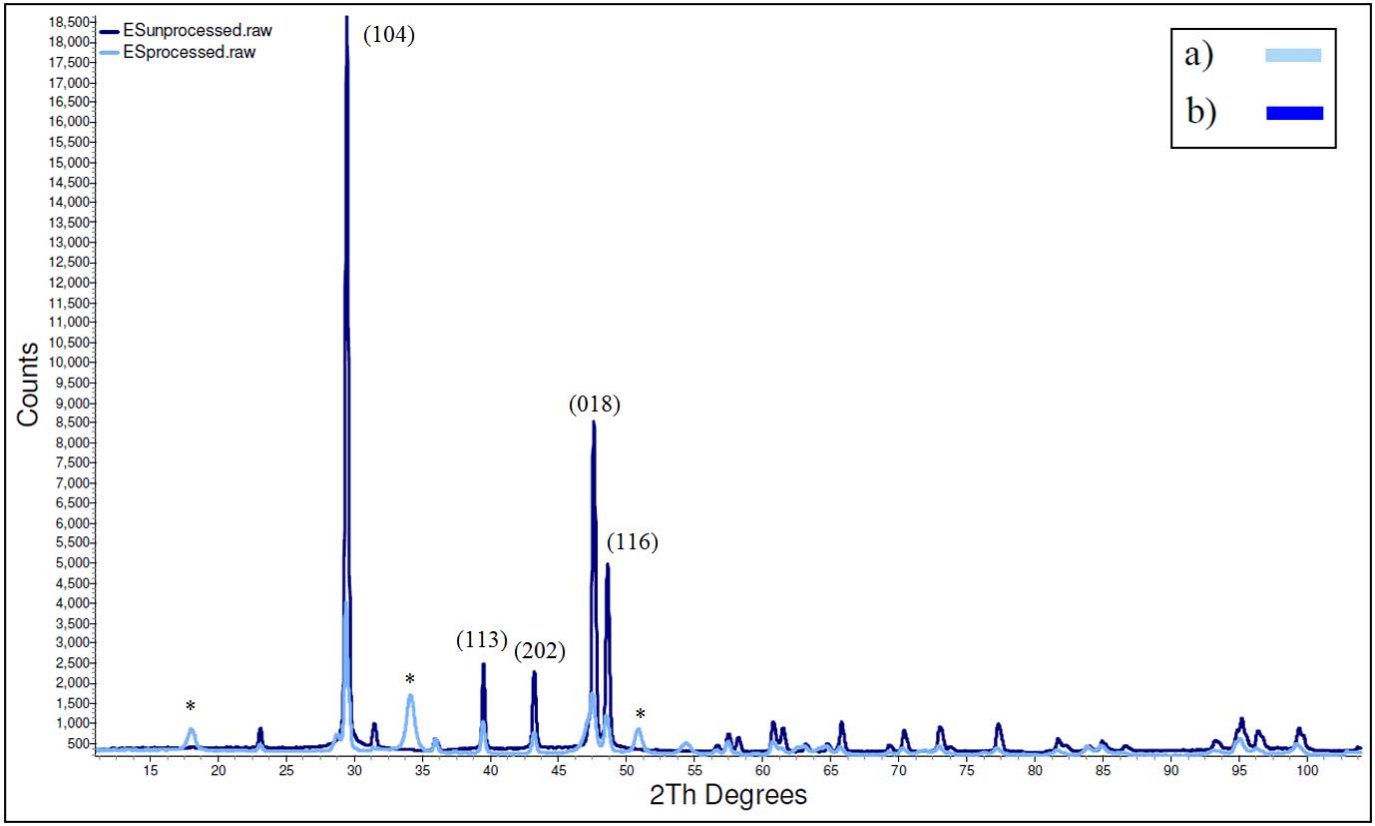
**X-ray Diffraction patterns of a) unprocessed eggshell, and b) nanofibrous structure generated on the eggshell substrate.** The peaks marked with asterisk (*) correspond to calcium hydroxide.

In order to observe the possible phase changes of nanofibrous structures as a result of laser irradiation, the XRD pattern of unprocessed eggshell (Figure. 5b) and the nanofibrous structures (Figure. 5a) can be compared. From Figure. 5 one can notice that the XRD patterns for both samples show a significant peak around 30°(2θ). This is the characteristic of crystalline calcite which indicates the *hkl *(104) [6,20]. However, there are three peaks marked with asterisks (*) which are associated with calcium hydroxide [21]. Laser irradiation might result in calcium carbonate decomposition to calcium oxide. This calcium oxide later would convert to calcium hydroxide due to atmosphere exposure [6,21]. Comparing Figures 5a and 5b, it can be observed that the intensity of the XRD peaks has been decreased for nanofibrous structures due to the reduction of the calcite crystal sizes.

Previous *in vitro *and *in vivo *studies have pointed out that the microporosity of the bone substitute surface structure as well as its biodegradability play an important role in bone healing. Thus, the generated nanofibrous structure with different porosity shows a different degree of biodegradability when implanted in the biological environment. Microporosity influences the bone substitute dissolution rate in biological fluids; hence a surface with higher porosity shows better degradability. Biodegradation of bone substitutes is vital to initiate the bone deposition process [22,23]. Porous structures increase adsorption of proteins such as bone morphogenetic proteins and other necessary ones required for bone formation which consequently influences cell adhesion and the subsequent cell proliferation and differentiation of osteoblasts [4,22,23]. On the other hand, cell attachment and proliferation are improved for nanostructures in comparison with micron-structures owing to higher effective surface area of the nanofibers [17]. As a result, we believe that the calcite nanofibrous structure generated on the eggshell substrate could enhance the biodegradability as well as the osteoconductivity of the surface in comparison with nanoparticles or micron-structure.

## Conclusion

This study describes a novel technique to synthesize calcium carbonate nanofibrous structure from eggshell using high repetition femtosecond laser under ambient condition. To the best of our knowledge, this is the first time that synthesizing 3D calcium carbonate nanofibrous structures using femtosecond laser have been reported. The morphological analyses by SEM and TEM were confirmed that fabricated nanofibers have approximately uniform 3D structure with average size of 50 nm. Further experiments showed that by changing the laser pulse repetition, different nanofibrous structure with different porosity could be achieved. The XRD and EDX analyses showed that laser irradiation barely affects chemical decomposition, though; part of the organic matter believes to be changed to calcium hydroxide owing to laser irradiation. This proposed method suggests a promising step in synthesizing interwoven 3D platforms from natural biomaterials to support new bone formation and achieve rapid bone healing as well as to improve develop new functional biomaterials for various biomedical applications. In vitro test to investigate the degradation rate of the nanofibrous scaffold in physiological environments and cell culture assays to understand the scaffold-cell interaction are being undertaken.

## Materials and methods

The avian eggshell representing 11% of the total weight of the egg consists mainly of calcium carbonate (94%), calcium phosphate (1%), organic matter (4%) and magnesium carbonate (1%) [6]. Hen's eggs were purchased, emptied and washed thoroughly with distilled water to get rid of dirt and organic layer.

**Figure 6:**
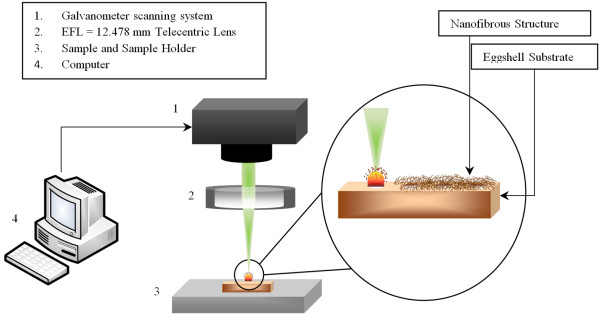
**Experimental Setup**.

Experiments were carried out with a 1040 nm wavelength direct-diode-pumped Yb-doped fiber amplified ultrafast laser system. Due to the solid state operation and high spatial mode quality of fiber lasers, Yb-doped fiber-oscillator/fiber-amplifier operates under low noise performance. Also, all the Laser parameters, such as laser repetition rate, pulse width and beam power are computer monitored which allows a precise interaction with the performed experiments. The schematic diagram of the experimental setup is depicted in Figure. 6. To investigate the effects of pulse repetition rate on morphology of generated nanofibrous structures, experiment were performed at laser repetition of 4, 8 and 13 MHz.

The nanofibrous structures were then characterized using Scanning Electronic Microscopy (SEM) followed by Energy dispersive X-ray spectroscopy (EDS) analyses. The nanoparticle aggregation and nanofiber size were analyzed by Transmission Electron Microscope (TEM). The samples were sonicated in isopropanol solution to separate the nanostructures from the substrate. Then a drop of the nanofiber-dispersed solution was placed on the copper grid and allowed to dry in a desiccator.

Phase analysis of the synthesized structures was performed using X-ray Diffraction (XRD). The x-ray source was a Cu rotating anode generator (Rigaku) with parallel focused beam and 3-circle diffractometer (Bruker D8) with a 2D detector (Bruker Smart 6000 CCD). The average wavelength of the x-rays was 1.54184Å. Phi scans with widths of 60°were done with the detector at four different swing angles for each sample in order to get a profile with a 2θ range of 10.5-104°.

## Competing interests

The authors declare that they have no competing interests.

## Authors' contributions

AT and KV conceived and designed the experimental strategy. AT performed the experiments, and wrote the manuscript. BT and KV helped with the editing the paper. All authors read and approved the final manuscript.
